# Identification of candidate genes associated with less-photosensitive anthocyanin phenotype using an EMS mutant (*pind*) in eggplant (*Solanum melongena* L.)

**DOI:** 10.3389/fpls.2023.1282661

**Published:** 2023-12-11

**Authors:** Lei Luo, Jos Molthoff, Qiang Li, Ying Liu, Shuangxia Luo, Na Li, Shuxin Xuan, Yanhua Wang, Shuxing Shen, Arnaud G. Bovy, Jianjun Zhao, Xueping Chen

**Affiliations:** ^1^ State Key Laboratory of North China Crop Improvement and Regulation, Key Laboratory of Vegetable Germplasm Innovation and Utilization of Hebei, Collaborative Innovation Center of Vegetable Industry in Hebei, International Joint R & D Center of Hebei Province in Modern Agricultural Biotechnology, College of Horticulture, Hebei Agricultural University, Baoding, China; ^2^ Plant Breeding, Wageningen University and Research, Wageningen, Netherlands; ^3^ Horticulture and Product Physiology, Wageningen University and Research, Wageningen, Netherlands

**Keywords:** eggplant, EMS mutants, anthocyanin, less-photosensitive, MutMap

## Abstract

Eggplant (*Solanum melongena* L.) is a highly nutritious and economically important vegetable crop. However, the fruit peel of eggplant often shows poor coloration owing to low-light intensity during cultivation, especially in the winter. The less-photosensitive varieties produce anthocyanin in low light or even dark conditions, making them valuable breeding materials. Nevertheless, genes responsible for anthocyanin biosynthesis in less-photosensitive eggplant varieties are not characterized. In this study, an EMS mutant, named *purple in the dark* (*pind*), was used to identify the key genes responsible for less-photosensitive coloration. Under natural conditions, the peel color and anthocyanin content in *pind* fruits were similar to that of wildtype ‘14-345’. The bagged *pind* fruits were light purple, whereas those of ‘14-345’ were white; and the anthocyanin content in the *pind* fruit peel was significantly higher than that in ‘14-345’. Genetic analysis revealed that the less-photosensitive trait was controlled by a single dominant gene. The candidate gene was mapped on chromosome 10 in the region 7.72 Mb to 11.71 Mb. Thirty-five differentially expressed genes, including 12 structural genes, such as *CHS*, *CHI*, *F3H*, *DFR*, *ANS*, and *UFGT*, and three transcription factors *MYB113*, *GL3*, and *TTG2*, were identified in *pind* using RNA-seq. Four candidate genes *EGP21875* (myb domain protein 113), *EGP21950* (unknown protein), *EGP21953* (CAAX amino-terminal protease family protein), and *EGP21961* (CAAX amino-terminal protease family protein) were identified as putative genes associated with less-photosensitive anthocyanin biosynthesis in *pind*. These findings may clarify the molecular mechanisms underlying less-photosensitive anthocyanin biosynthesis in eggplant.

## 1 Introduction

Anthocyanins are natural flavonoid pigments, which are responsible for blue, purple and red colors in plant tissues, such as flowers, fruits, seed coats, leaves, and stems. In addition to their role in coloring, anthocyanins attract pollinators and seed disperser and protect plants from biotic and abiotic stresses (Fan et al., 2016; Sivankalyani et al., 2016; Sun et al., 2018; Naing and Kim, 2021). Anthocyanins also show beneficial effects on human health, including antioxidation, antimutagenicity, cardiovascular disease prevention, liver protection, and the inhibition of tumor cell metastasis (Pojer et al., 2013; Liu et al., 2016).

Anthocyanins are produced through the phenylpropanoid pathway, which has been studied in many plants, such as petunia and *Arabidopsis* (Krol et al., 1990; Dooner et al., 1991; Timothy and Holton, 1995; Baudry et al., 2004). The anthocyanin biosynthesis is catalyzed stepwise by a series of enzymes, including phenylalanine ammonia-lyase (PAL), cinnamate-4-hydroxylase (C4H), 4-coumarate coenzyme A ligase (4CL), chalcone synthase (CHS), chalcone isomerase (CHI), flavanone 3-hydroxylase (F3H), flavonoid 3′-hydroxylase (F3′H), flavonoid 3′,5′-hydroxylase (F3′5′H), dihydroflavonol 4-reductase (DFR), anthocyanidin synthase (ANS), and UDP-glucose: flavonoid-3-O-glycosyl transferase (UFGT). The expression of these structural genes was primarily regulated by three classes of transcription factors (MYB, bHLH, and WD40), which always formed a MYB-bHLH-WD40 (MBW) complex. (Gonzalez et al., 2008; Albert et al., 2014; Xu et al., 2015; Shan et al., 2019; Li et al., 2020). Among them, MYB TFs have been identified to be the major determinant regulator in the MBW complex (Gonzalez et al., 2008; Tang et al., 2020). In *Arabidopsis*, MYB TFs TT2 (TRANSPARENT TESTA 2), PAP1/PAP2 (the production of anthocyanin pigment 1/2), MYB113 and MYB114, bHLH TFs TT8 (transparent testa8), and GL3 (glabra3), WD40 repeat protein TTG1 (TRANSPARENT TESTA GLABRA1) are the main components of the MBW complexes that regulate anthocyanin production (Zhang et al., 2003; Baudry et al., 2004).

The biosynthesis of anthocyanins is often affected by environmental factors such as light (Sun et al., 2020; Zhao et al., 2022) and temperature (Gao-Takai et al., 2019; Ryu et al., 2020). Most plants accumulate anthocyanin in a light-induced manner (Meng and Liu, 2015; Kim et al., 2021; Ma et al., 2021a), and anthocyanin concentration tends to rise as the light intensity increase (Li et al., 2018; Bai et al., 2019; Hong et al., 2019). In light signal transduction, phytochromes and their downstream factors, such as COP1 (CONSTITUTIVE PHOTOMORPHOGENIC1), HY5 (LONG HYPOCOTYL 5), and other TFs participate in light-induced anthocyanin biosynthesis (Maier et al., 2013; Meng and Liu, 2015; Hoai Nguyen, 2020; Bhatia et al., 2021; Ma et al., 2021b). In this biological process, expression of structural genes and regulatory genes, except WD40, is strongly dependent upon the existence of light (Takos et al., 2006; Bai et al., 2017; Hong et al., 2019; Liu et al., 2019). Interestingly, less- and non-photosensitive biosynthesis of anthocyanins has been reported in cherry, chrysanthemum, mango, turnip, grape and eggplant (Huang et al., 2016; Yang et al., 2017; Guo et al., 2018; He et al., 2019; Zha et al., 2019; Shi et al., 2021), although the underlying mechanism is still unclear.

Eggplant (*Solanum melongena* L.) is a globally cultivated vegetable crop with high economic benefits (Saini and Kaushik, 2019; Oladosu et al., 2021). The color of eggplant fruit is an important quality trait, with purple varieties exhibiting high levels of anthocyanins in the fruit peel (Nayanathara et al., 2016; Nino-Medina et al., 2017). However, low light during cultivation often leads to poor coloration, which reduces their visual quality and commercial value (Li et al., 2018). Therefore, eggplant genotypes with less- and non-photosensitive anthocyanin biosynthesis are valuable for the breeding of low-light tolerant varieties. The purple peel under the calyx is a good indicator of less- and non-photosensitive coloration (Xiang et al., 2015; He et al., 2019). Although some quantitative trait loci (QTL) that are responsible for purple peel under calyx have been identified in eggplant (Chen, 2015; Toppino et al., 2016; Mangino et al., 2022) and markers have been developed (Xiang et al., 2015; Zhang et al., 2021b), the underlying causal genes conferring less-photosensitive biosynthesis of anthocyanin in eggplant remain unknown.

In this study, a less-photosensitive anthocyanin biosynthesis mutant *purple in the dark* (*pind*) in eggplant was discovered. Under bagging conditions, the *pind* fruit color was light purple, whereas the wild type was white, indicating that anthocyanin biosynthesis in the *pind* mutant was less dependent on light. Phenotypic and genetic analysis, gene mapping, and transcriptomic analysis were performed to identify the causative genes conferring less-photosensitive anthocyanin biosynthesis in the fruit peel of *pind*. Taken together, our results provided novel insight into less-photosensitive anthocyanin accumulation in eggplant fruit peel.

## 2 Materials and methods

### 2.1 Materials and population construction

**Figure 1:**
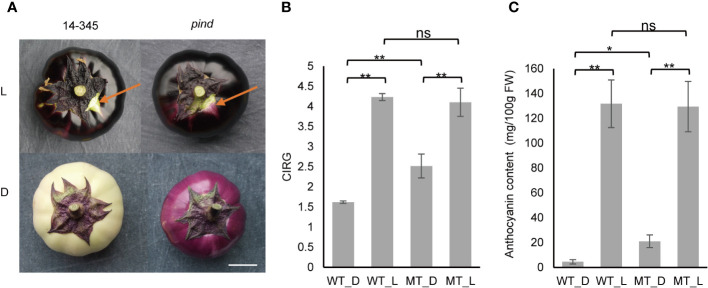
Effect of bagging on the peel color of ‘14-345’ (wild type) and *pind* (mutant) fruits. **(A)** Fruit color of ‘14-345’ (WT) and *pind* (MT) under natural light (L) and dark (D) conditions. The peel color under the calyx is indicated with arrows. White bar = 4cm. **(B)** CIRG (color index of red grape) value and **(C)** total anthocyanin content of the peel of ‘14-345’ and *pind* under natural and dark conditions. Data marked with *, ** and ns indicate *P* <0.05, *P*<0.01 and no significance, respectively.

The mutant was identified from M_1_ line in an ethyl methanesulfonate (EMS) mutagenized population of eggplant variety ‘14-345’, which is white under the calyx. After two generations of self-fertilization, we obtained a homozygous mutant and named it “*pind”* (**Figure 1A**). The *pind* mutant and wildtype ‘14-345’ were crossed to produce F_1_ plants. Subsequently, F_2_ and BC_1_ populations were generated from F_1_ plants via self- and backcross with ‘14-345’ or *pind*, respectively. They were used as segregating populations for genetic analysis and mapping the candidate gene. In addition, *pind* and a cultivated eggplant ‘18-305’ (**Figure S1**), which is white under the calyx, were crossed to obtain another F_2_ population (‘305F_2_’). The ‘305F_2_’ population was also used for gene mapping. All plants were grown in a plastic greenhouse in the experimental fields of Hebei Agricultural University, Baoding, China. During the cultivation, the temperature inside the greenhouse was ranged from 18 to 32°C, the air humidity ranged from 50 to 70%, and the daytime light intensity ranged from 40k to 55k lx.

### 2.2 Phenotypic investigation and measurement of anthocyanin content and color index

The fruit of the plants were bagged on the 5^th^ day after flowering and the fruit color and anthocyanin content were investigated on the 14^th^ day under bagging condition. Color differences were assessed by the color index of red grapes (CIRG). A CR-400 colorimeter (Konica Minolta, Chroma Meter, Osaka, Japan) was used to measure the values of L*, a*, and b*. CIRG was calculated with the equation CIRG = [(180-*H*)/(L* + C*)], *H*=arctan (b*/a*), *C*=(a*^2^ + b*^2^)^0.5^ (Carreno et al., 1995; Zhang et al., 2008).

The total anthocyanin content in the peel was quantified using the pH differential spectroscopic method (Cheng and Patrick, 1991).

One-way analysis of variance (ANOVA) was conducted on the CIRG value and anthocyanin content, and significant differences between groups were assessed by Duncan’s multiple range tests (p < 0.05) using SPSS 16.0 Statistics (SPSS Inc., Chicago, IL, USA).

### 2.3 Whole genome sequence of bulked DNA

In the F_2_ population under bagging conditions, 30 plants with purple fruit and 30 plants with white fruits were selected to construct the mixed pools ‘P’ and ‘G’, respectively. Genome DNA was extracted using the CTAB method.

Bulk segregant analysis (BSA)-based sequencing and MutMap analysis were used to map the candidate genes. The qualified DNA was randomly broken into fragments with a length of 350bp, and libraries were built using a TruSeq Library Construction Kit, followed by Illumina PE150 sequencing. BWA software was used to map the clean data to the eggplant reference genome (Li et al., 2021). The UnifiedGenotyper module of GATK3.8 software (Mckenna et al., 2010) was used to detect SNPs, and VariantFiltration was used to filter SNP detection. The SNP-index value with ‘14-345’ as the reference was calculated to identify the key genes. The candidate intervals were determined with the threshold value: SNP-index (P) > 0.67, SNP-index (G) < 0.1, and Δ(SNP-index) > 0.6.

### 2.4 Obtaining recombinants

To narrow down the candidate region, the genotype of individuals in the F_2_ and ‘305F_2_’ populations was detected using Kompetitive allele specific PCR (KASP) technology to screen recombinants. The primers used for genotype verification of the individual plants are listed in **Table S1**.

### 2.5 RNA extraction, library construction, and RNA-sequencing

Total RNA was extracted using an Eastep Super Kit (Shanghai Promega, Shanghai, China). The extracted RNA was treated with DNase to remove the genome DNA and the integrity and quantity were examined using 1% agarose gels and a Nanodrop 2000c Spectrophotometer (Thermo Nanodrop Technologies, Wilmington, DE, USA). A total of 1 μg RNA per sample was used to construct RNA-seq libraries. After qualification, mRNA was isolated from total RNA using oligo-magnetic beads. The mRNA was interrupted, reverse transcribed into cDNA, and then the cDNA was purified with an AMpure XP system (Beckman Coulter, Beverly, MA, USA). Sequencing libraries were generated using NEBNext^®^ Ultra™ RNA Library Prep Kit for Illumina^®^ (NEB, Boston, MA, USA). Twenty-four libraries were sequenced on the Illumina Hiseq platform (Illumina, San Diego, CA, USA). The library construction and sequencing were completed by Novogene (Beijing, China).

### 2.6 RNA sequencing data analysis

Raw data (raw reads) in fastq format were filtered to obtain clean reads, and the Q20, Q30, and GC content of the clean data were calculated. The clean data were aligned to the eggplant reference genome (Li et al., 2021) using Hisat2 v2.0.5. software (Mortazavi et al., 2008). The featureCounts v.5.0-p3 tool in the subread software was used to count the reads mapped to each gene (Liao et al., 2014) and the expected number of fragments per kilobase of transcript sequence per millions base pairs sequenced (FPKM) value was calculated based on the read count and the length of the gene. The differential expression analysis was performed using the DESeq2 R package (1.20.0), and the genes with the criteria |log_2_(fold change)| > 1 and padj≤ 0.05 were considered DEGs (Love et al., 2014). The relative expression level log_2_ (ratios) of all DEGs was clustered by the K-means method. Plant TFs were predicted by hmmscan based on iTAK software (Perez-Rodriguez et al., 2010). Kyoto Encyclopedia of Genes and Genomes (KEGG) enrichment was analyzed with clusterProfile software and a padj of less than 0.05 was considered significantly enriched (Yu et al., 2012).

### 2.7 Quantitative real-time PCR analysis

Fourteen genes were chosen for the validation of RNA-seq using qRT-PCR. Primer Premier 5.0 software was used to design the primers, which are listed in **Table S2**. A total of 1 μg RNA per sample was reverse transcribed using a PrimeScript™ reagent Kit with gDNA Eraser (TaKaRa, Beijing, China) in 20 μL of reaction mixture. The qRT-PCR was performed on a LightCycler^®^ 96 instrument (Roche, Basel, Switzerland), using THUNDERBIRD SYBR qPCR Mix (TOYOBO, Shanghai, China) with the following reactions: 95°C 2 min; 95°C 30s, 60°C 10s, and 68°C 10s for 40 cycles. The PCR products were quantified by 2^−△△CT^ method (Livak and Schmittgen, 2001) with normalization to the expression level of *SmGAPDH* (*EGP1067575*). Significant differences between groups were assessed by Student’s ***t***-test (p < 0.05) using SPSS 16.0 Statistics (SPSS Inc., Chicago, IL, USA).

## 3 Results

### 3.1 Phenotypic characterization of *pind*

The *pind* mutant was obtained from eggplant ‘14-345’ mutagenized population with EMS. The coloration of the ‘14-345’ fruit peel is photosensitive with white-colored fruit under the calyx. Interestingly, a light purple pigmentation was observed under the calyx of *pind*, indicating that the coloration of *pind* was less photosensitive. To further characterize the less-photosensitive coloration in *pind*, the fruit was bagged to mimic dark conditions. Compared to the white fruit of ‘14-345’, the *pind* fruit was light purple (**Figure 1A**). Under natural conditions, the color index, CIRG value, of ‘14-345’ was similar to that of *pind*, whereas the CIRG value of the *pind* mutant was significantly higher than that of ‘14-345’ under bagging conditions (**Figure 1B**).

The anthocyanin contents in the peel of ‘14-345’ and *pind* were also quantified. The anthocyanin content in the peel of ‘14-345’ and *pind* was comparable under natural conditions. However, the level of anthocyanin in the bagged eggplants decreased significantly in both ‘14-345’ and *pind* compared with natural conditions. Anthocyanin content in the peel of bagged *pind* (21.1 mg/100 g FW) was significantly higher than that in the bagged ‘14-345’ (4.5 mg/100 g FW) (**Figure 1C**). These results indicated that light was required for the coloration of fruit peel in ‘14-345’, but less so for *pind*.

### 3.2 Genetic analysis of *pind*

To investigate the inheritance of *pind*, segregating populations were constructed. All the F_1_ plants from the crosses of *pind* and ‘14-345’ had purple fruits under bagging conditions, which was phenocopied *pind*. Out of the F_2_ plants, 116 had purple bagged fruits, while 37 had white bagged fruits. A chi-squared test revealed that this segregation pattern agreed with the 3:1 Mendelian segregation ratio (P=0.82>0.05, χ^2^ = 0.05<χ^2^_0.05,1_ = 3.84). In the W-BC_1_ population (the backcross progeny of F_1_ with ‘14-345’), 30 plants displayed white wild type fruit, whereas the remaining 23 plants produced purple fruit under bagging conditions. The segregation pattern fitted the ratio 1:1 (P=0.34>0.05, χ^2^ = 0.93<χ^2^_0.05,1_ = 3.84). All M-BC_1_ plants, the backcross progeny of F_1_ with *pind*, exhibited purple fruits under bagging conditions (**Table 1**). These results suggested that the mutant gene was a single dominant allele.

**Table 1:** The segregation of the fruit color in dark condition.

Generation	Numbers of plants	*pind* phenotype	WT phenotype	Expected ratio	P	χ^2 c^
14-345	15	0	15			
*pind*	45	45	0			
F_1_	14	14	0			
F_2_	153	116	37	3:1	0.82	0.05
M-BC_1_ ^a^	59	59	0			
W-BC_1_ ^b^	53	23	30	1:1	0.34	0.93

a M-BC_1_ represents the backcross progeny of F_1_ with *pind*.

b W-BC_1_ represents the backcross progeny of F_1_ with 14-345.

c χ^2^_0.05_ = 3.84, df=1.

### 3.3 Identification of the candidate genomic region

**Figure 2:**
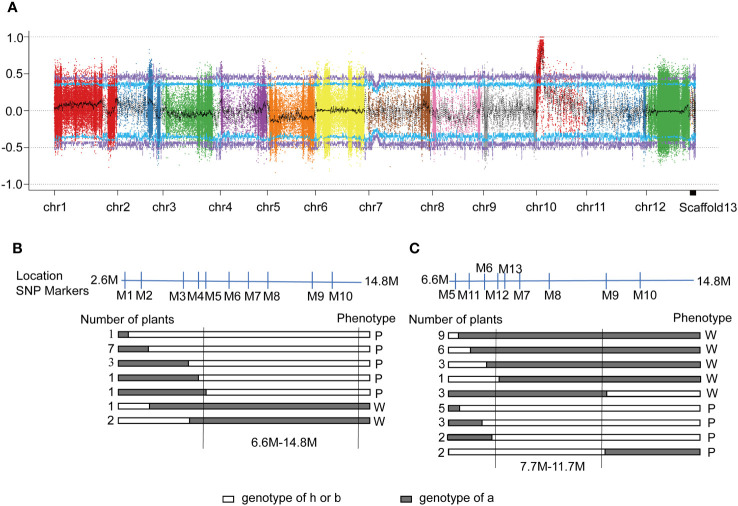
Mapping and identification of the candidate gene. **(A)** The distribution of Δ(SNP-index) on all chromosomes. X-axis: Chromosome ID; Y-axis: Δ(SNP-index). Each colored dot represents an Δ(SNP-index) value of an SNP site. Schematic representation of allelic segregation of recombinants in F_2_. **(B)** and ‘305F_2_’ **(C)**. a: Genotype of wildtype ‘14-345’ **(B)** or ‘18-305’ **(C)**; b: Genotype of the *pind* mutant; h: Heterozygote of ‘14-345’ and *pind*. P: Purple fruit peel under bagging conditions; W: White fruit peel under bagging conditions.

To identify the genomic region responsible for less-photosensitive anthocyanin biosynthesis in the fruit peel of *pind*, BSA-based genome resequencing and MutMap analysis were performed. Quality control resulted in 96,777,013 and 90,196,644 high-quality reads and 98.67% and 98.64% of them were mapped on the eggplant genome (**Tables S3, S4**), from which 1421190 SNPs were detected in total. Based on the Δ(SNP-index), a single significant interval in a 12.2 Mb region (from 2.6 to 14.8 Mb) on chromosome 10 was identified as a candidate region of the mutation (**Figure 2A**). There were 2,058 SNPs, including 22 non-synonymous mutation sites.

To identify the causal mutation in this region, several molecular markers were designed to uniformly cover the preliminary mapping interval. Fifteen recombinant plants were identified and the candidate gene was mapped to an 8.2 Mb region (from 6.6 Mb to 14.8 Mb) between markers M5 and M10 (**Figure 2B**).

To further map the SNP related to less-photosensitive anthocyanin biosynthesis, another F_2_ population, ‘305F_2_’ (*pind* × ‘18-305’), was used to obtain recombinants. The ‘305F_2_’ population consisted of 408 plants, of which 305 had purple fruit and 103 had white fruit under bagging conditions (**Table S5**). Thirty-four recombinants were identified and the candidate gene was finally mapped to a 4.0 Mb region between the markers M12 and M9 (**Figure 2C**), from 7.7 Mb~11.7 Mb on chromosome 10. A total of 224 SNPs that were associated with 75 genes (**Table S6**), were detected in the candidate interval.

### 3.4 Transcriptome comparison between *pind* and ‘14-345’ in response to dark treatment

**Figure 3:**
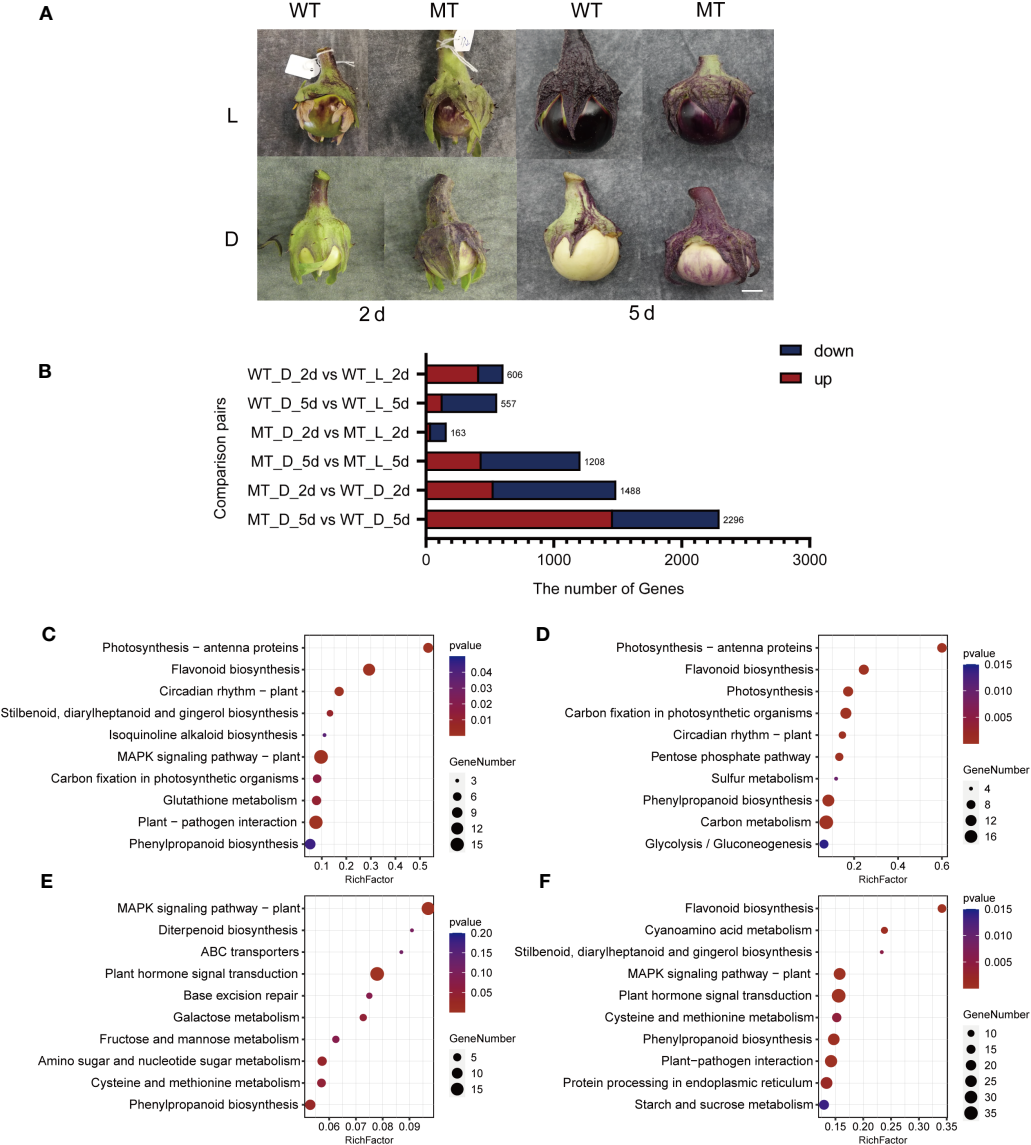
Overview of DEGs identified by RNA-seq analysis in the peel of ‘14-345’ and *pind* fruits under bagging conditions at two developmental stages. **(A)** The fruit color of ‘14-345’(WT) and *pind* (MT) under natural (L) and dark (D) conditions at 2 days (2d) and 5 days (5d) under bagging conditions. Scale bar represents 1 cm. **(B)** The number of DEGs between samples in each combination. **(C–F)** KEGG pathway enrichment of DEGs in ‘WT_D_2d vs. WT_L_2d’, ‘WT_D_5d vs.WT_L_5d’, ‘WT_D_2d vs. MT_D_2d’, and ‘WT_D_5d vs. MT_D_5d’.

To identify the candidate gene responsible for less-photosensitive anthocyanin biosynthesis in *pind*, RNA-seq analysis was performed on the fruits of *pind* (MT) and ‘14-345’ (WT) under bagging (D) and natural (L) conditions for two stages, 2days (2d) and 5 days (5d) after bagging. For both stages, the fruits of the ‘14-345’ bagged were white, whereas the fruits of *pind* were light purple under bagged conditions. The fruit peel color of ‘14-345’ and *pind* at the two stages was almost the same under natural conditions (**Figure 3A**).

To identify the key differentially expressed genes (DEGs) between the fruit peel of ‘14-345’ and *pind*, pairwise comparisons between bagged ‘14-345’ and *pind* at each stage (MT_D_2d vs. WT_D_2d, MT_D_5d vs. WT_D_5d), bagged and natural ‘14-345’ at each stage (WT_D_2d vs. WT_L_2d, WT_D_5d vs. WT_L_5d), and bagged and natural *pind* at each stage (MT_D_2d vs. MT_L_2d, MT_D_5d vs. MT_L_5d) were analyzed. A total of 4,338 DEGs were detected in the above-mentioned six combinations (**Figure 3B**).

A total of 415 and 191 genes were significantly induced and inhibited in the peel of ‘14-345’ under bagging conditions on 2d, respectively, compared with natural conditions (WT_D_2d vs. WT_L_2d). KEGG enrichment analysis showed that the pathways ‘flavonoid biosynthesis’ and ‘phenylpropanoid biosynthesis’ were significantly enriched (**Figure 3C**). There were 557 DEGs in the ‘WT_D_5d vs. WT_L_5d’ combination, in which the pathways ‘flavonoid biosynthesis’ and ‘phenylpropanoid biosynthesis’ were also significantly enriched (**Figure 3D**). The decreased expression of the eight flavonoid biosynthetic genes, including *CHS* (*EGP24357*, *EGP16216*), *CHI* (*EGP24232*, *EGP22200*), *F3H* (*EGP30923*), *F3′5′H* (*EGP32037*), *DFR* (*EGP31016*), and *ANS* (*EGP18904*) (**Figures S2A, B**), indicated that the anthocyanin biosynthetic pathway was repressed in ‘14-345’ under dark conditions.

A total of 1,488 DEGs were detected in the ‘MT_D_2d vs. WT_D_2d’ combination, which were significantly enriched in the ‘MAPK signal pathway’ and ‘plant signal transduction pathway’ (**Figure 3E**). The ‘MT_D_5d vs. WT_D_5d’ combination produced 1,463 DEGs, and the enrichment analysis revealed that among all of the pathways identified, the ‘flavonoid biosynthesis pathway’ were the most significantly enriched (**Figure 3F**). The expression of structural genes *4CL* (*EGP10904*), *CHS* (E*GP24357*), *CHI* (*EGP24232*, *EGP22200*), *F3H* (*EGP30923*), *F3′5′H* (*EGP32037*), *DFR* (*EGP31016*), and *ANS* (*EGP18904*) (**Figure S2C**) involved in anthocyanin biosynthesis was significantly higher in the mutant than in the wild type at 5d under bagging conditions.

### 3.5 Analysis of DEGs for less-photosensitive anthocyanin biosynthesis in *pind*

**Figure 4:**
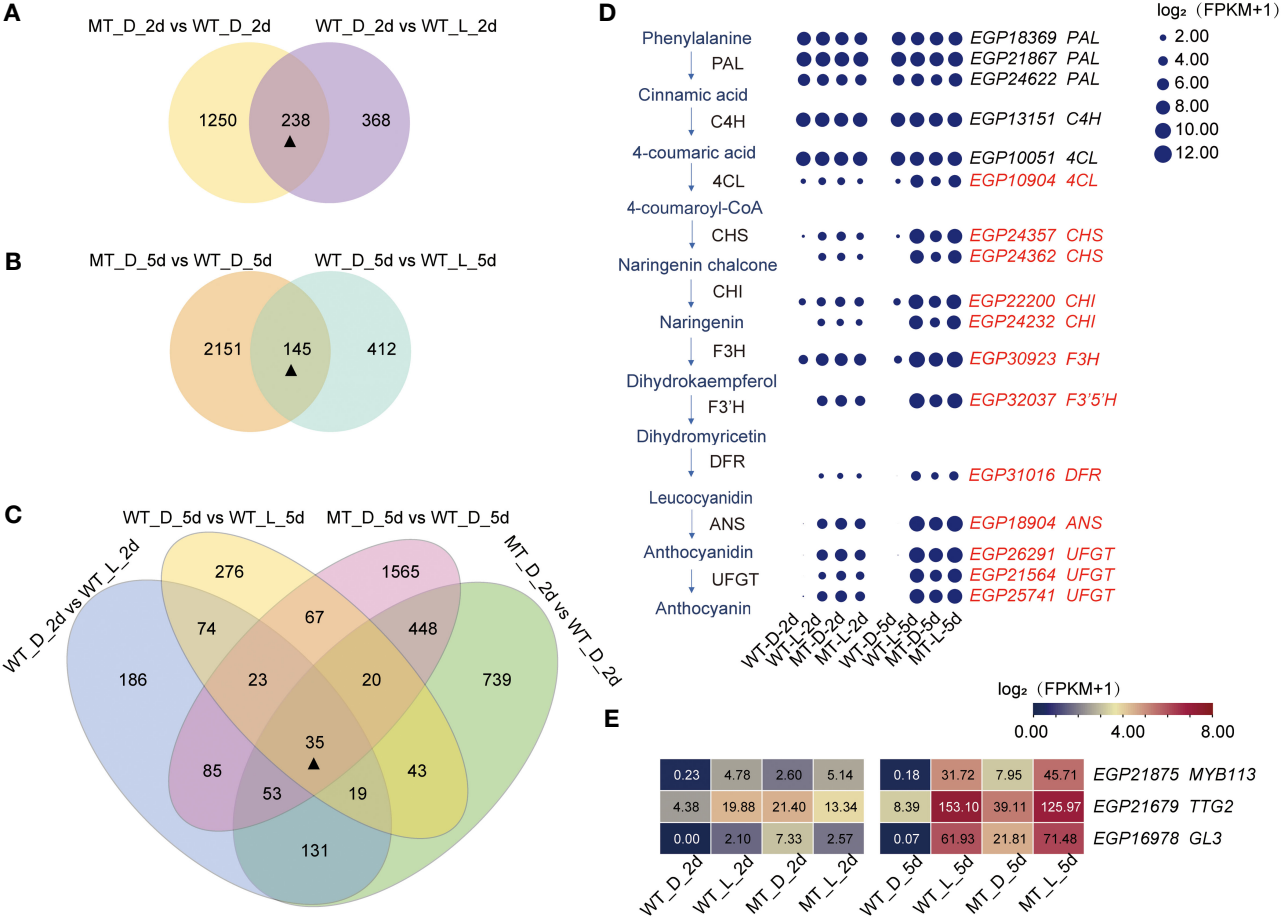
Identification of the DEGs related to less-photosensitive anthocyanin biosynthesis in *pind.* **(A–C)** Venn diagram of the different comparisons of DEGs. **(D)** Expression pattern analysis of structural genes involved in less-photosensitive anthocyanin biosynthesis in fruit peel of *pind*. Note: Red gene ID represents candidate DEGs related to less-photosensitive anthocyanin biosynthesis in the fruit peel of *pind*. The size of the circle represents log_2_ (FPKM+1); **(E)** Expression pattern analysis of TFs involved in less-photosensitive anthocyanin biosynthesis in the fruit peel of *pind*. The number in the box indicates the FPKM value.

Due to the difference in fruit color between ‘14-345’ and *pind* under bagged conditions (MT_D vs. WT_D) and the differences in the color of ‘14-345’ fruit between bagged and natural conditions (WT_D vs. WT_L), we screened for common DEGs in these combinations. The Venn diagram clearly illustrated the relationships of DEGs between different comparisons, and 238 DEGs (**Figure 4A**; **Table S7**) and 145 DEGs (**Figure 4B**; **Table S8**) were commonly shared by ‘MT_D vs. WT_D’ and ‘WT_D vs. WT_L’ on 2d and 5d, respectively. Moreover, 35 DEGs were commonly shared by these four combinations (**Figure 4C**; **Table S9**), which may participate in anthocyanin biosynthesis in *pind* under dark conditions. Therefore, these 35 DEGs, including 12 structural genes and three TFs, were used for further analysis.

Under natural conditions, there was no significant difference in the expression of structural genes between ‘14-345’ and *pind* fruit peel. Bagging inhibited the transcripts of most of the structural genes, including *4CL*, *CHS*, *CHI*, *F3H*, *F3′5′H*, *DFR*, and *ANS* in ‘14-345’ while not in *pind*, as the transcriptional level of structural genes were extremely higher in *pind* than in ‘14-345’ (**Figure 4D**). These data suggest that ‘14-345’ and *pind* have different responses to bagging in terms of anthocyanin biosynthesis. The highly expressed structural genes in *pind* under dark conditions showed *pind* was less photosensitive.

TFs can regulate the expression of structural genes by directly binding to *cis*-regulatory elements in the promoter of the genes, which play important roles in plant growth and development (Chen et al., 2019; Jiang et al., 2022; Liu et al., 2023). In this study, MYB TF *SmMYB113* (*EGP21875*), WRKY TF *SmTTG2* (*EGP21679*), and bHLH TF *SmGL3* (*EGP16978*) were identified from the 35 DEGs as the putative main regulators of the anthocyanin biosynthesis in a less-photosensitive manner in the fruit peel of *pind*. Under natural conditions, the expression of *SmMYB113*, *SmTTG2*, and *SmGL3* was comparable between ‘14-345’ and *pind*. Under bagging conditions, *SmMYB113*, *SmTTG2*, and *SmGL3* was down-regulated both in ‘14-345’ and *pind* compared to natural conditions (**Figure 4E**). Notably, the expression levels of *MYB113*, *TTG2*, and *GL3* were significantly increased in the peel of *pind* compared to ‘14-345’ under bagged conditions (**Figure 4E**).

**Figure 5:**
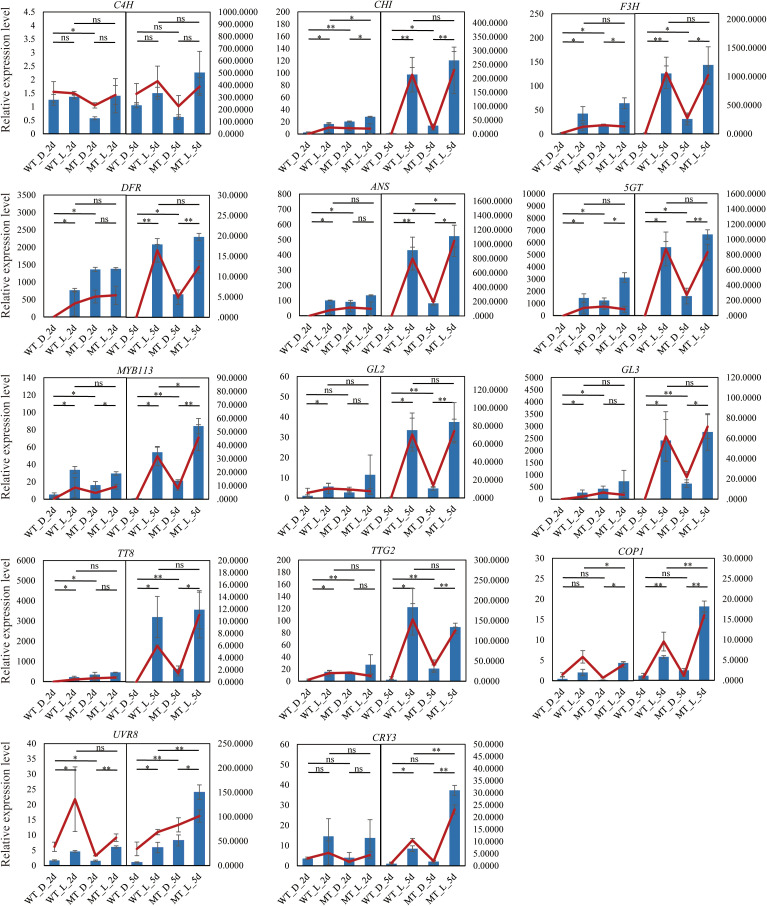
Validation of RNA-seq results using qRT-PCR. Y-axis: relative expression. The blue bar and red line represent the data from qRT-PCR and RNA-seq data, respectively. Asterisks indicate significant differences determined by the Student’s *t*-test (*P < 0.05, **P < 0.01). ns, no significance.

To confirm the reliability of the RNA-seq data, six structural genes (*C4H*, *CHI*, *F3H*, *DFR*, *ANS*, and *5GT*), five TFs (*MYB113*, *GL2*, *GL3*, *TT8*, and *TTG2*), and three genes involved in light signaling (*COP1*, *UVR8*, and *CRY3*) were selected to analyze expression profiles by qRT-PCR. The transcript abundances of these genes determined using qRT-PCR agreed with those determined from transcriptome sequencing (**Figure 5**), indicating the reliability of the RNA-seq results.

### 3.6 Candidate gene analysis

**Figure 6:**
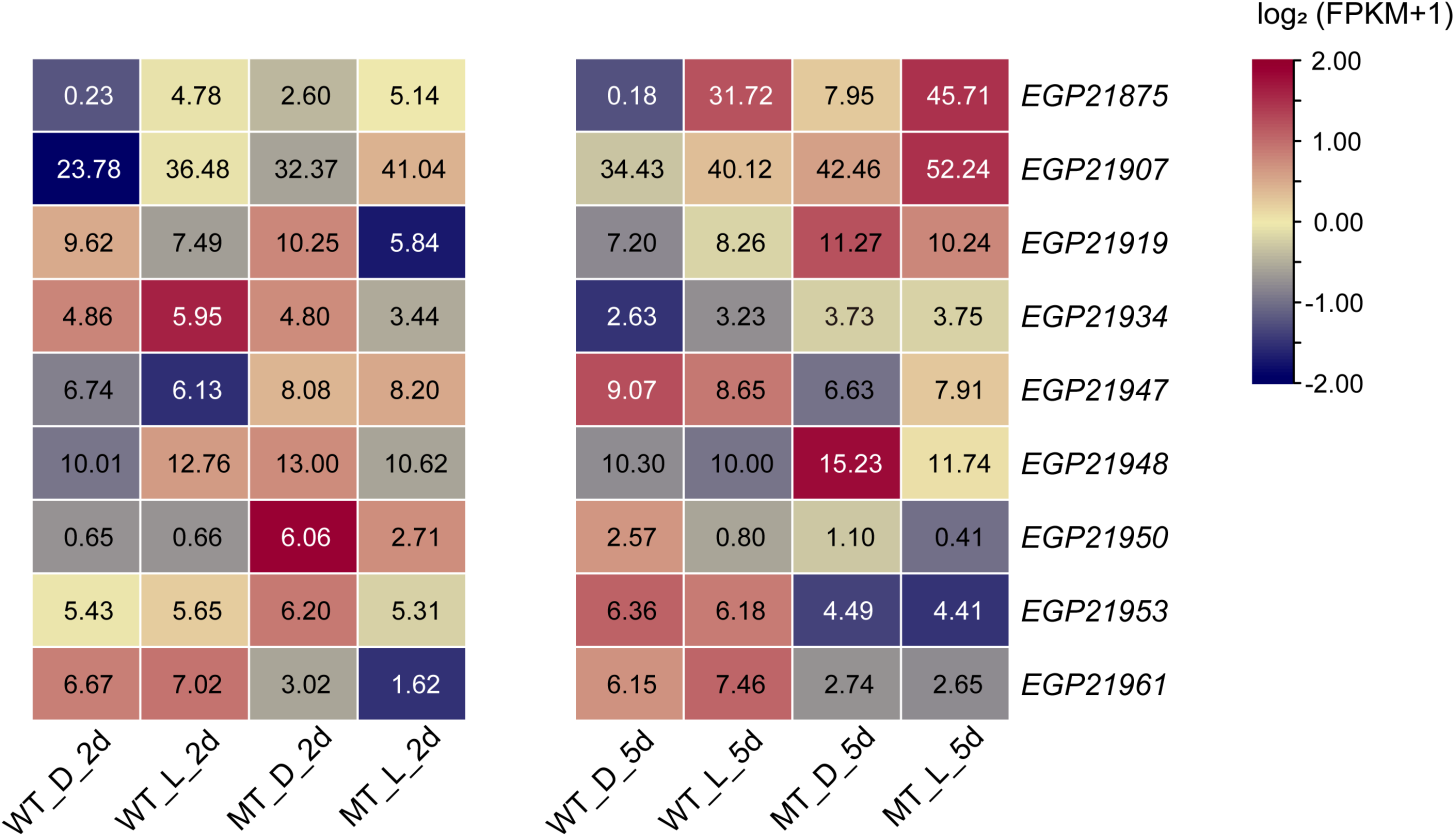
Expression pattern analysis of the genes in the candidate region. The number in the box indicates the FPKM value.

Based on the gene mapping analysis, there were 75 genes in the candidate region (**Table S6**), of which only nine (**Figure 6**) were expressed in the eggplant peel according to the transcriptome analysis. Twenty-seven SNPs were detected to be associated with these nine genes, and only one non-synonymous SNP (within *EGP21953*) was identified. The gene *EGP21953* (**Table 2**) had an amino acid residue substitution at aa-262 (Ala-to-Tyr mutation). According to the annotated information, *EGP21953* encodes a CAAX amino-terminal protease family protein. However, there was no significant difference in *EGP21953* expression between ‘14-345’ and *pind*, neither in natural nor in bagged conditions.

**Table 2:** Candidate genes related to less-photosensitive anthocyanin synthesis in the fruit peel of *pind*.

Gene ID	SNP loci	substitution type	SNP category	Distance	Predicted function
*EGP21875*	7808145 7770881	C->A T->C	Intergenic region in upstream, Intergenic region in downstream	22893bp 13006bp	myb domain protein 113
*EGP21950*	10889680	T->C	Upstream	859bp	–
*EGP21953*	11268541	C->T	Non-synonymous		CAAX amino terminal protease family protein
*EGP21961*	11685483	T->A	Intergenic region in upstream	6998bp	CAAX amino terminal protease family protein

Among the other eight genes, whose related SNPs were detected in intergenic regions or introns, only *EGP21875*, *EGP21950*, and *EGP21961*, were differentially expressed between the peel of *pind* and ‘14-345’ (**Figure 6** **;** **Table 2**). *EGP21875* encodes an MYB TF, “*SmMYB113*”, which has been shown to participate in the regulation of anthocyanin biosynthesis (Zhang et al., 2014; Jiang et al., 2016; He et al., 2019; Yang et al., 2022). The expression of *SmMYB113* was inhibited in the fruit peel of ‘14-345’ under bagged conditions. Interestingly, *SmMYB113* showed significantly higher expression in the peel of *pind* than in ‘14-345’ at both 2d and 5d stages, pinpointing the activation of SmMYB113 in *pind* in darkness. There was one SNP 22.9 kb upstream of the start codon and one SNP 13 kb downstream of the stop codon of *SmMYB113*, respectively. Because the intergenic SNPs play a potentially important role in phenotype variation in plants by altering gene expression (Schwartz et al., 2009; Ding et al., 2012; Li et al., 2012; Wang et al., 2015), these two SNPs in the intergenic region associated with *SmMYB113* may affect its expression.

*EGP21950* encoded an unknown functional protein, and there was one SNP 859 bp upstream of the start codon of *EGP21950*. The expression of *EGP21950* was significantly up-regulated in the fruit peel of *pind* in comparison to ‘14-345’ at 2d under bagged conditions, but its expression level showed no significant difference between ‘14-345’ and *pind* under natural conditions and at 5d under bagged conditions. In addition to *EGP21953*, *EGP21961* also encoded a CAAX amino-terminal protease family protein, and its associated SNP was about 7 kb upstream of the initiation codon. *EGP21961* was down-regulated significantly in the peel of *pind* compared to ‘14-345’, in both natural and bagged conditions.

## 4 Discussion

### 4.1 Gene identification related to less-photosensitive anthocyanin biosynthesis in eggplant

Anthocyanin is an important flavonoid type pigment in flowers and fruits, with light being an essential regulator of anthocyanin accumulation (Takos et al., 2006; Meng and Liu, 2015; Bai et al., 2017; Kim et al., 2021; Ma et al., 2021a). Proper anthocyanin accumulation in fruits or flowers can be hindered by low-light conditions (caused by weather and climate, the cultivation facility, or plant morphology) (Hong et al., 2015; Cao et al., 2016; Zhang et al., 2021a; Guo et al., 2022). Therefore, understanding the mechanism of less-and non-photosensitive anthocyanin biosynthesis and creating the less- and non-photosensitive cultivars will provide valuable resources for the breeding of low-light tolerant varieties

In this study, we discovered a less-photosensitive anthocyanin biosynthesis mutant (*pind*) in eggplant, and a candidate gene that was physically mapped to 7.7 ~11.7 Mb on chromosome 10 (**Figure 2C**). This result was consistent with previous reports, showing that the candidate genes that may correspond to loci controlling fruit peel color under calyx in eggplants were mapped to chromosome 10 (Zhang et al., 2021b; Mangino et al., 2022; Qiao et al., 2022). In addition to chromosome 10, the candidate genes controlling the fruit peel color under calyx in eggplants were also found on other chromosomes, including chromosome 1, 3, 4, and 11 (Mangino et al., 2022; Qiao et al., 2022). However, the above studies did not distinguish materials as less-photosensitive or non-photosensitive materials. When comparing the phenotypic differences between the less-photosensitive or non-photosensitive materials, it was observed that under bagging conditions, fruit coloration became significantly lighter for less-photosensitive materials (**Figure 1A**), whereas there was no significant difference for non-photosensitive materials (He et al., 2019; He et al., 2022). Recent studies have found that the candidate gene related to non-photosensitive anthocyanin biosynthesis in eggplant is *SmFTSH10* (He et al., 2022), which was physically close to, but outside the candidate interval 7.7Mb-11.7Mb (**Figure 3C**) for less-photosensitive anthocyanin biosynthesis in this study, indicating less- and non-photosensitive anthocyanin biosynthesis in eggplant may be controlled by different locus.

### 4.2 Candidate genes related to less-photosensitive anthocyanin biosynthesis in *pind*

Through MutMap and transcriptome analysis, we obtained four candidate genes: *EGP21875*, *EGP21950*, *EGP21953*, *and EGP21961* (**Table 2**). *EGP21953* and *EGP21961* are homologs of the gene *AT1G14270.1* in *Arabidopsis*, which is a CAAX amino-terminal protease family member. However, at present, there is no literature or report indicating that the CAAX amino-terminal protease family proteins under investigation exert regulatory control over anthocyanin biosynthesis. *EGP21950* encodes a protein with an unknown function, that contained a DUF616 (Protein of unknown function) domain (**Figure S3**). Additionally, proteins encoded by structural genes and regulatory genes didn’t possess CAAX prenyl endopeptidase-like domains or UDF616 domains (**Figure S3**), indicating their low likelihood to bind with CAAX amino-terminal protease family proteins or DUF616 proteins. Therefore, we speculate that *EGP21950*, *EGP21953*, or *EGP21961* had minimal potential in contributing to the genetic basis of less-photosensitivity.

The gene *EGP21875* encoded an MYB TF SmMYB113, homologous to *AtMYB113* in *Arabidopsis*. In this study, *SmMYB113* was hardly expressed in the fruit peel of wild type ‘14-345’ under bagged conditions, whereas its expression was considerably up-regulated in the *pind* mutant compared to ‘14-345’. It was reported that overexpression of *SmMYB113* (also known as *SmMYB1*) resulted in the up-regulation of *SmCHS*, *SmCHI*, *SmF3H*, *SmANS*, and other genes, and a high level of anthocyanin accumulation (Zhang et al., 2014; Yang et al., 2022). Thus, anthocyanin-pigmentation in *pind* in the dark may be related to the mutations of *SmMYB113*. Collectively, we speculated that *EGP21875* (*SmMYB113*) was the best candidate gene for the regulation of less-photosensitive anthocyanin biosynthesis in the fruit peel of *pind*.

In addition, the SNPs related to *EGP21875* (*SmMYB113*) were more than 13kb from the coding region. Some studies have shown that a non-coding region far from the gene promoter may change the activity of neighboring genes. For example, the *Teosinte Branched 1* (*TB1*) gene is mainly responsible for a major-effect QTL that controls morphological differences in plant architecture between maize (*Zea mays* subsp. *mays*) and its wild relative, teosinte (*Z. mays* subsp. *mexicana* and subsp. *parviglumis*) (Clark et al., 2004; Studer et al., 2011); the expression of *tb1* was altered by a sequence >41kb upstream of *tb1* (Clark et al., 2006). In addition, *booster1* (*b1*) was a regulator gene responsible for the biosynthesis of flavonoid pigments in maize; a 6-kb region at about 100kb upstream of the transcription start site of gene *b1* was found to be a hepta-repeat enhancer, which may increase the transcription initiation rate (Stam et al., 2002). The non-coding region located distally from the gene promoter could be considered as a putative *cis*-regulatory element that modulates gene expression through recruitment of transcription factors (Stadhouders et al., 2014; Visser et al., 2014; Roberts et al., 2016). Studies have shown that natural variation in *cis*-regulatory regions of genes played important roles in phenotypic variations by altering gene expression (Frary et al., 2000; Schwartz et al., 2009; Muños et al., 2011; Van Der Knaap et al., 2014; Chu et al., 2019). Taken together, we speculated that the position of the SNPs in the intergenic region of *EGP21875* (*SmMYB113*) might coincide with a regulatory element that controls the *SmMYB113* expression level.

### 4.3 The activation of MYB113 may be necessary for anthocyanin accumulation in *pind* peel under dark conditions

**Figure 7:**
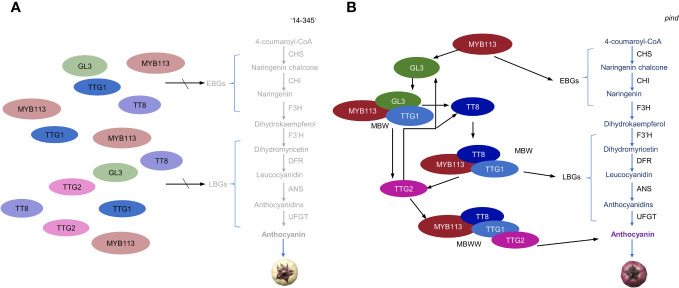
A hypothetical model of the mechanism for anthocyanin biosynthesis in the eggplant peel of ‘14-345’ **(A)** and *pind* **(B)** under dark conditions. **(A)** Under bagging conditions, in ‘14-345’, the inactivated state of transcription factors, SmMYB113, SmGL3, SmTTG2 and SmTT8, cannot promote the expression of structural genes, consequently resulting in the inhibition of structural gene expression and subsequently impacting the biosynthesis of anthocyanins. **(B)** TF MYB113 promotes the expression of EBGs and activates the expression of *GL3*. MYB113, and GL3 form a MBW complex with the WD40 protein TTG1 to promote the expression of *TT8*. Then MYB113, TT8, and TTG1 will form a new MBW complex to promote the expression of LBGs. *TTG2* is activated by MBW and forms the MBWW complex, and the MBWW complex promotes the expression of genes related to anthocyanin transport. The highly expressed structural genes in *pind* under dark conditions led to accumulation of anthocyanin. The positive regulation of genes is marked in black arrows. The gray font represents genes with very low expression, while the lighter-colored circles indicate transcription factors with low activity.

Under bagging conditions, ‘14-345’ fruits exhibited white coloration while the *pind* mutants displayed purple coloration. Consequently, under bagging conditions, the structural genes were expressed at a minimal level in the peel of ‘14-345’ but showed higher expression in *pind* (**Figure 4D**). It is well known that the expression of structural genes is directly regulated by transcription factors, among which MYB TFs, bHLH TFs, and WD40 are key modulators (Shan et al., 2019; Li et al., 2020). In this study, expression of *SmMYB113*, *SmGL3* (bHLH) and *SmTTG2* (WRKY) was significantly higher in *pind* peel than ‘14-345’ in darkness (**Figure 4E**). Moreover, bHLH TF *SmTT8* was inactive in ‘14-345’ under bagged conditions, but strongly activated in *pind* on 5d (**Table S8**; **Figure 5**). Therefore, under bagging conditions, the inactive transcription factors, such as SmMYB113, SmGL3, SmWRY44 and SmTT8, cannot stimulate the expression of structural genes, which is the underlying reason for the inability of ‘14-345’ to produce anthocyanins under bagging conditions (**Figure 7A**).

In *Arabidopsis*, AtMYB113 interacts with AtTT8 or AtGL3 and AtTTG1, forming the MBW complex, to regulate the expression of late biosynthesis genes (LBGs) (Ramsay and Glover, 2005). The MYB TF is the main determinant of the MBW complex, and MYBs control the expression of early biosynthesis genes (EBGs) and LBGs (Stracke et al., 2007; Gonzalez et al., 2008; Tang et al., 2020). In tomato, after the overexpression of *SlANT1* (a homolog of *AtMYB113*), the expression of the structural genes, and the bHLH gene *SlAN1* (a homolog of *AtTT8*) was significantly up-regulated (Mathews et al., 2003; Kiferle et al., 2015). SlAN1 probably activates the expression of structural genes through the MYB-AN1-WD40 complex, whereas SlJAF13 (a homolog of AtGL3) regulates the transcription of *SlAN1* through the MYB-SlJAF13-WD40 complex (Montefiori et al., 2015; Liu et al., 2018). Therefore, we speculated that under bagged conditions, the up-regulation of EBGs was mainly due to the activation of SmMYB113 in the peel of *pind*; the LBGs were directly regulated by the MBW complex composed of SmMYB113, SmTT8, and WD40 (SmTTG1), whereas the expression of *SmTT8* was activated by the MBW complex SmMYB113-SmGL3-SmTTG1; and *SmGL3* was activated by SmMYB113 (**Figure 7B**).

In addition, WRKY44 was reported to regulate anthocyanin accumulation. In tobacco, the coloration of flowers was observed to increase upon overexpression of *TTG2*, whereas a decrease in flower coloration was achieved through silencing *TTG2* (Li et al., 2017). Anthocyanin accumulation was induced in tobacco leaves after transient overexpression of kiwifruit *WRKY44* (Peng et al., 2020). WRKY44 was reported to interact with BMW to regulate the corresponding biological processes (Pesch et al., 2014; Gonzalez et al., 2016; Verweij et al., 2016; Lloyd et al., 2017). In summary, it was suggested that under bagging conditions, the expression of *SmTTG2* was up-regulated by the MBW complex and SmTTG2 could bind to the MBW complex to promote the expression of structural genes in *pind* (**Figure 7B**).

Based on the expression patterns and putative function of the genes in other plants, we proposed a working model that describes the regulatory mechanism of anthocyanin biosynthesis in the peel of ‘14-345’ and *pind* under dark conditions (**Figure 7**). It is indicated that the activation of *SmMYB113*, *SmTT8*, *SmTTG2* and *SmTT8* is the necessary factor for anthocyanin accumulation in the dark in eggplant. Considering that SmMYB113 was the most upstream in the pathway, it is postulated that SmMYB113 was the first transcription factor to be activated and serves as the most crucial regulator. Combined with gene mapping analysis, it is speculated that the activation of *SmMYB113* in *pind* under bagged conditions may be related to the SNP of the intergenic region of *EGP21875*.

## Data availability statement

The original contributions presented in the study are publicly available. This data can be found here: https://www.ncbi.nlm.nih.gov/bioproject/PRJNA1014581, https://www.ncbi.nlm.nih.gov/bioproject/PRJNA1013920.

## Author contributions

LL: Conceptualization, Investigation, Visualization, Writing – original draft, Writing – review & editing. JM: Conceptualization, Methodology, Software, Writing – original draft. QL: Conceptualization, Visualization, Writing – review & editing. YL: Conceptualization, Methodology, Writing – review & editing. SL: Data curation, Investigation, Writing – review & editing. NL: Visualization, Writing – review & editing. SX: Visualization, Writing – review & editing. YW: Visualization, Writing – review & editing. SS: Funding acquisition, Supervision, Writing – review & editing. AB: Conceptualization, Supervision, Writing – review & editing. JZ: Funding acquisition, Supervision, Writing – review & editing. XC: Data curation, Funding acquisition, Supervision, Writing – review & editing.
