# Supplementary figures and images for: Identification of candidate genes associated with less-photosensitive anthocyanin phenotype using an EMS mutant (pind) in eggplant (Solanum melongena L.)
Source: Front Plant Sci. 2023 Dec 11;14:1282661. doi: 10.3389/fpls.2023.1282661 (PMC10758619; doi:10.3389/fpls.2023.1282661)

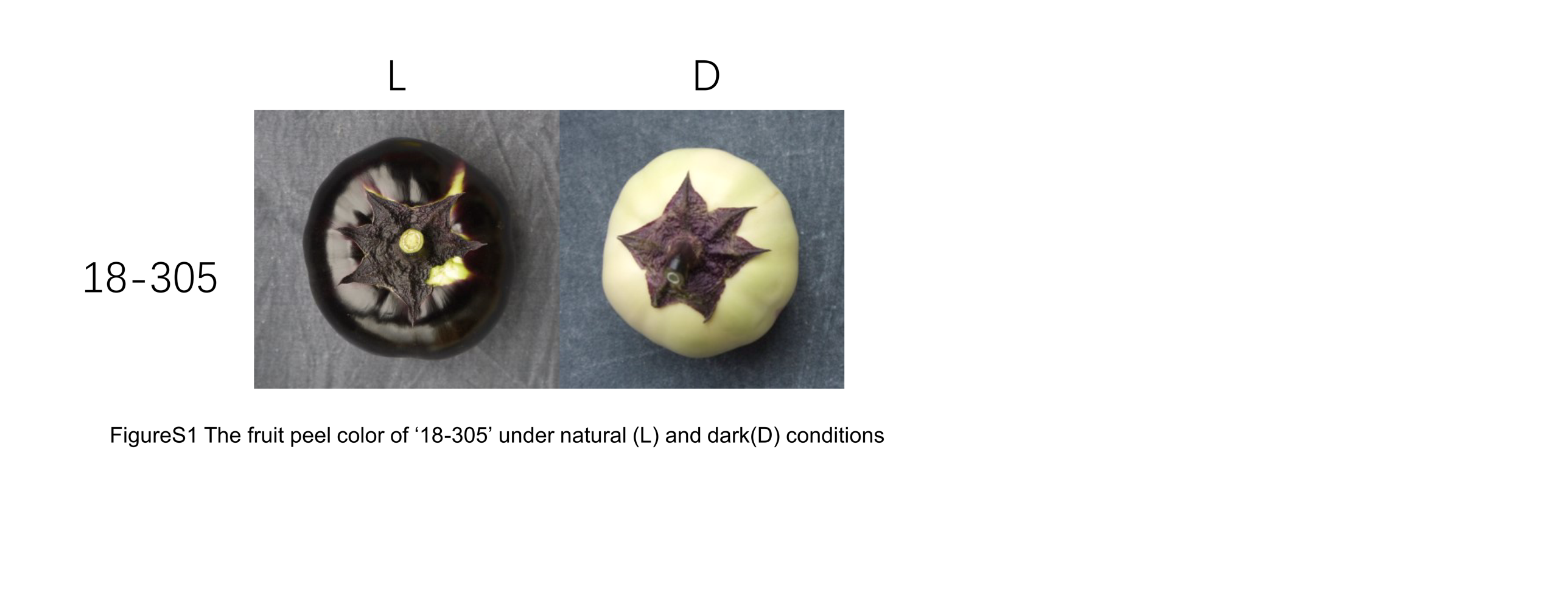

Supplement: Supplementary file 1 [file Image_1.tif]

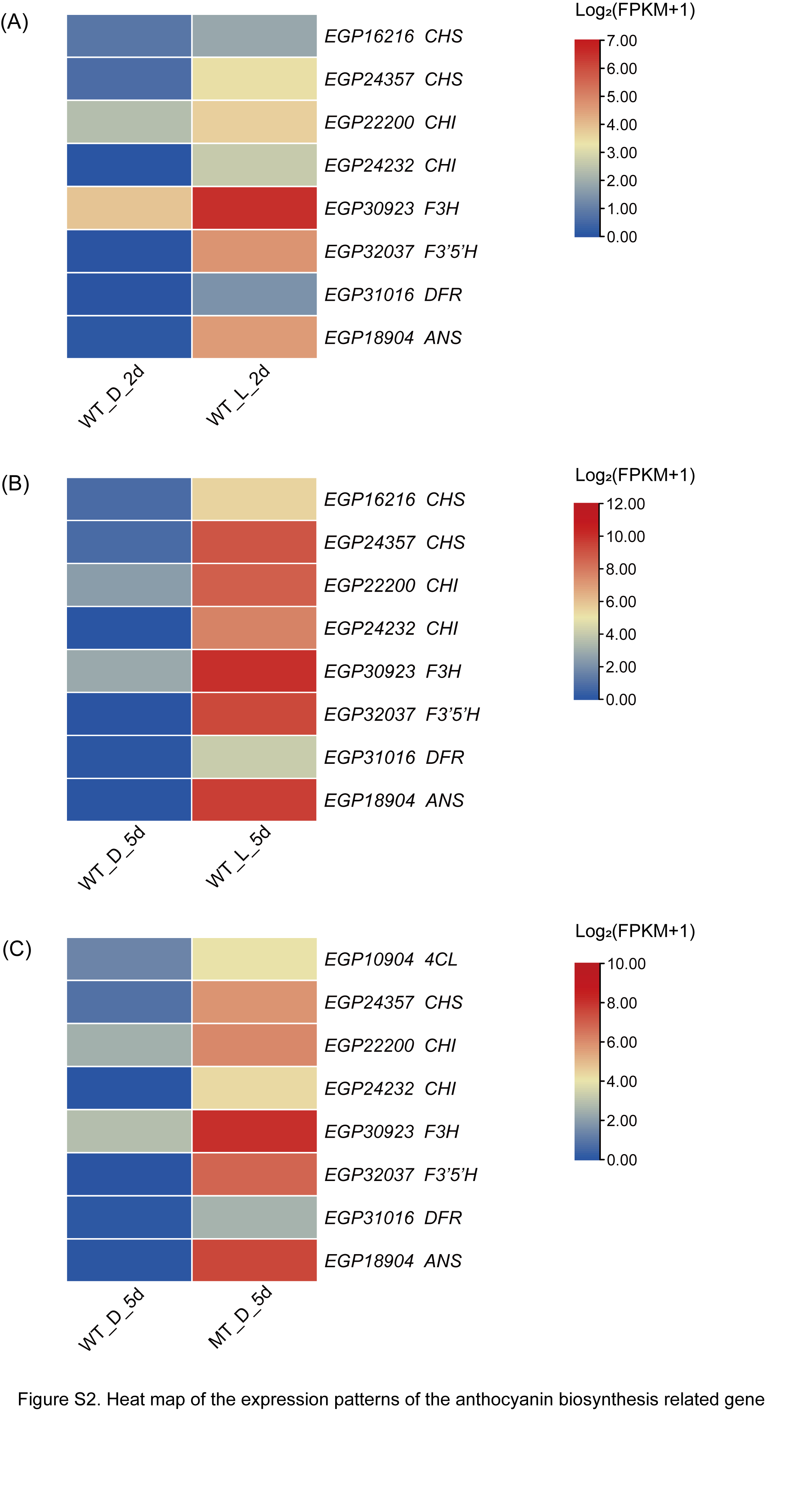

Supplement: Supplementary file 2 [file Image_2.tif]
